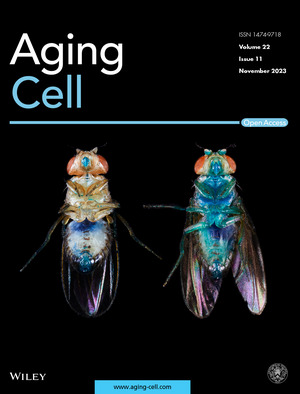# Featured Cover

**DOI:** 10.1111/acel.14045

**Published:** 2023-11-16

**Authors:** Flaminia Zane, Hayet Bouzid, Sofia Sosa Marmol, Mira Brazane, Savandara Besse, Julia Lisa Molina, Céline Cansell, Fanny Aprahamian, Sylvère Durand, Jessica Ayache, Christophe Antoniewski, Nicolas Todd, Clément Carré, Michael Rera

## Abstract

Cover legend: The cover image is based on the Research Article *Smurfness‐based two‐phase model of ageing helps deconvolve the ageing transcriptional signature* by Flaminia Zane et al., https://doi.org/10.1111/acel.13946 Image Credit: Michael Rera and Aurore Colibert